# Normal on the Inside: A Case of Recurrent Abdominal Pain, Equivocal CT Appendicitis, and Histologically Normal Appendix in an Adolescent With Chronic Cannabis Use

**DOI:** 10.7759/cureus.117214

**Published:** 2026-09-30

**Authors:** Nazar Abdelgabar, Yahia Khalil, Karrar Al-Balootti, Raya Abu-Khalaf, Mohammad Z Khalil, Layan Abu-Khalaf

**Affiliations:** 1 Gastroenterology and Hepatology, St Mary’s Hospital, Isle of Wight, GBR; 2 General Surgery, St Mary's Hospital, Isle of Wight, GBR; 3 Neurosurgery, Salford Royal NHS Foundation Trust, Salford, GBR; 4 Medicine, Royal College of Surgeons in Ireland, Dublin, IRL; 5 Cardiology, Royal College of Surgeons in Ireland-Bahrain, Busaiteen, BHR; 6 Psychiatry, Blackpool Victoria Hospital, Blackpool, GBR

**Keywords:** acute appendicitis in adults, cannabinoid hyperemesis syndrome (chs), cyclic vomiting, diagnostic anchoring, normal appendix histology

## Abstract

Cyclic vomiting syndrome (CVS) and cannabinoid hyperemesis syndrome (CHS) are diagnoses of exclusion, and both can anchor clinical thinking in ways that delay recognition of organic pathology. We describe an 18-year-old male patient who attended the emergency department repeatedly between December 2025 and June 2026 with severe abdominal pain and vomiting, against a background of chronic cannabis use. Working diagnoses of CHS and CVS were recorded across multiple admissions. During his March 2026 admission, contrast-enhanced CT of the abdomen and pelvis (CTAP) showed a mildly thickened appendix with small-volume pelvic free fluid, reported as mild acute appendicitis. Conservative management was commenced but failed to resolve symptoms. Laparoscopic appendicectomy was performed. Intraoperatively, the appendix appeared mildly dilated and injected. Histopathological analysis returned a normal appendix vermiformis with no inflammation, dysplasia, or malignancy. The patient was discharged with resolution of his acute symptoms but re-presented within weeks with identical complaints. At the time of writing, oesophagogastroduodenoscopy and familial Mediterranean fever testing are pending. Faecal calprotectin was 159 microg/g. When CT findings, operative appearances, and histology point in different directions, the underlying diagnosis is not resolved by surgery; it has to be reconsidered from the beginning.

## Introduction

Acute appendicitis is among the most common indications for emergency abdominal surgery worldwide [1]. The classical presentation of migratory periumbilical pain localising to the right iliac fossa (RIF) with anorexia, nausea, and vomiting is well-described, but a substantial minority present atypically with diffuse pain, predominant vomiting, or absent peritoneal signs, and these are the presentations in which diagnosis is delayed [2]. Computed tomography (CT) of the abdomen and pelvis with intravenous contrast has a pooled sensitivity of approximately 97% and specificity of approximately 96% in adults and is the imaging modality of choice when the clinical picture is uncertain [3]. Its introduction into routine practice changed both operative decision-making and the use of hospital resources [4]. The diagnosis nonetheless remains histopathological, and a proportion of appendicectomy specimens contain no inflammation. In a United Kingdom (UK) multicentre observational study of emergency appendicectomy, a histologically normal appendix was removed in 20.6% of patients, with wide variation between centres [5].

Cyclic vomiting syndrome (CVS) is defined by recurrent, stereotyped episodes of severe nausea and vomiting separated by symptom-free intervals. The Rome IV criteria require exclusion of structural, metabolic, neurological, and gastrointestinal causes before the diagnosis is applied [6,7]. Cannabinoid hyperemesis syndrome (CHS) sits within the same framework and is defined by stereotypical episodic vomiting arising after prolonged heavy cannabis exposure, with resolution following sustained abstinence [8,9]. Neither condition has a confirmatory diagnostic test, and each depends on clinical history and the systematic exclusion of organic disease [10].

Chronic cannabis use alongside an established functional label creates conditions that favour diagnostic anchoring, the tendency to fix on an early working diagnosis and fail to revise it as new information emerges [11]. When CT then suggests mild appendicitis in such a patient, the decision to operate or to keep observing is difficult, and the histological result carries considerable weight in how the episode is understood afterwards.

We describe a case in which CT findings consistent with mild appendicitis led to appendicectomy in a young man whose recurrent vomiting had been attributed to CVS and CHS. Histopathological analysis of the resected appendix showed no inflammation. His symptoms continued after surgery, and the underlying diagnosis was still unresolved at the time of writing.

## Case presentation

The patient was an 18-year-old man who had been fit and well before this illness. He smoked cigarettes, drank alcohol occasionally, and used cannabis regularly. He worked as a cleaner and lived with his mother. He had no known drug allergies and took no regular medication. His family history included left ventricular enlargement in his biological father from adolescence, myocardial infarction in his maternal grandfather in his 50s, and ulcerative colitis in a maternal aunt and a maternal uncle.

From July 2025, he made several emergency department attendances and had one paediatric inpatient admission for recurrent crushing chest pain on the left side, which had been present for approximately two weeks when he first presented. Troponin was normal on each occasion, and the electrocardiogram (ECG) showed no ischaemic change, aside from an early finding of possible T wave inversion with a right bundle branch block that was attributed to a normal variant. Cardiology reviewed him in October 2025 and applied a Holter monitor over 24 hours. The cardiology documentation noted normal sinus rhythm, no significant arrhythmia, and normal PR and QTc intervals and concluded that the pain was not of cardiac origin. Cannabis and smoking cessation were advised.

On Day 1, he attended the emergency department with sudden onset of severe epigastric and umbilical pain that he rated 10 out of 10, with repeated vomiting and retching and loose stool. Examination revealed severe tenderness in the umbilical, right upper quadrant, and epigastric regions, with guarding. Laboratory values at this and subsequent attendances are given in Table 1. CT of the abdomen and pelvis showed no acute pancreatitis and no cholecystitis. The appendix was not visualised. Minimal pelvic free fluid and subcentimetre reactive mesenteric nodes were present, and no cause for his symptoms was identified (Figure 1). He was discharged with a diagnosis of suspected gastritis and prescriptions for codeine, hyoscine butylbromide, and omeprazole.

**Table 1:** Laboratory values at key attendances across the clinical course Reference ranges are those used by the reporting laboratory at Isle of Wight NHS Trust. Values outside the reference range are shown in bold. Values not documented at a given attendance are shown as not recorded. The Day 100 column corresponds to the attendance at which CT was reported as showing mild acute appendicitis

Parameter	Reference range	Day 0	Day 2	Day 98	Day 99	Day 100	Day 138	Day 186
Haemoglobin (g/L)	130-170	145	149	148	139	136	140	169
White cell count (x10⁹/L)	4.0-10.0	16.9	13.8	16.1	16.9	11.2	17.3	13.2
Neutrophils (x10⁹/L)	2.0-7.0	14.9	11.2	13.8	15.4	9.2	15.1	10.3
Platelets (x10⁹/L)	150-410	179	175	208	172	153	188	185
C reactive protein (mg/L)	0-5	2.3	8.6	3.2	4.6	5.3	1.5	1.2
Sodium (mmol/L)	133-146	143	142	140	139	141	141	139
Potassium (mmol/L)	3.5-5.3	3.5	3.8	3.6	3.6	3.4	3.2	3.0
Urea (mmol/L)	2.6-7.5	6.1	3.8	4.1	3.8	4.0	3.7	6.6
Creatinine (µmol/L)	58-99	69	69	77	60	71	67	82
Estimated glomerular filtration rate (mL/min/1.73 m²)	>90	>90	>90	>90	>90	>90	>90	>90
Bilirubin (µmol/L)	0-20	17	20	14	23	Not recorded	38	43
Alanine aminotransferase (U/L)	10-24	33	28	21	18	Not recorded	31	22
Alkaline phosphatase (U/L)	59-168	114	108	112	106	Not recorded	110	115
Albumin (g/L)	35-50	47	47	49	47	Not recorded	53	54
Amylase (U/L)	<100	75	Not recorded	103	76	Not recorded	61	71
International normalised ratio	0.8-1.2	Not recorded	1.0	1.0	Not recorded	Not recorded	Not recorded	1.1

**Figure 1:**
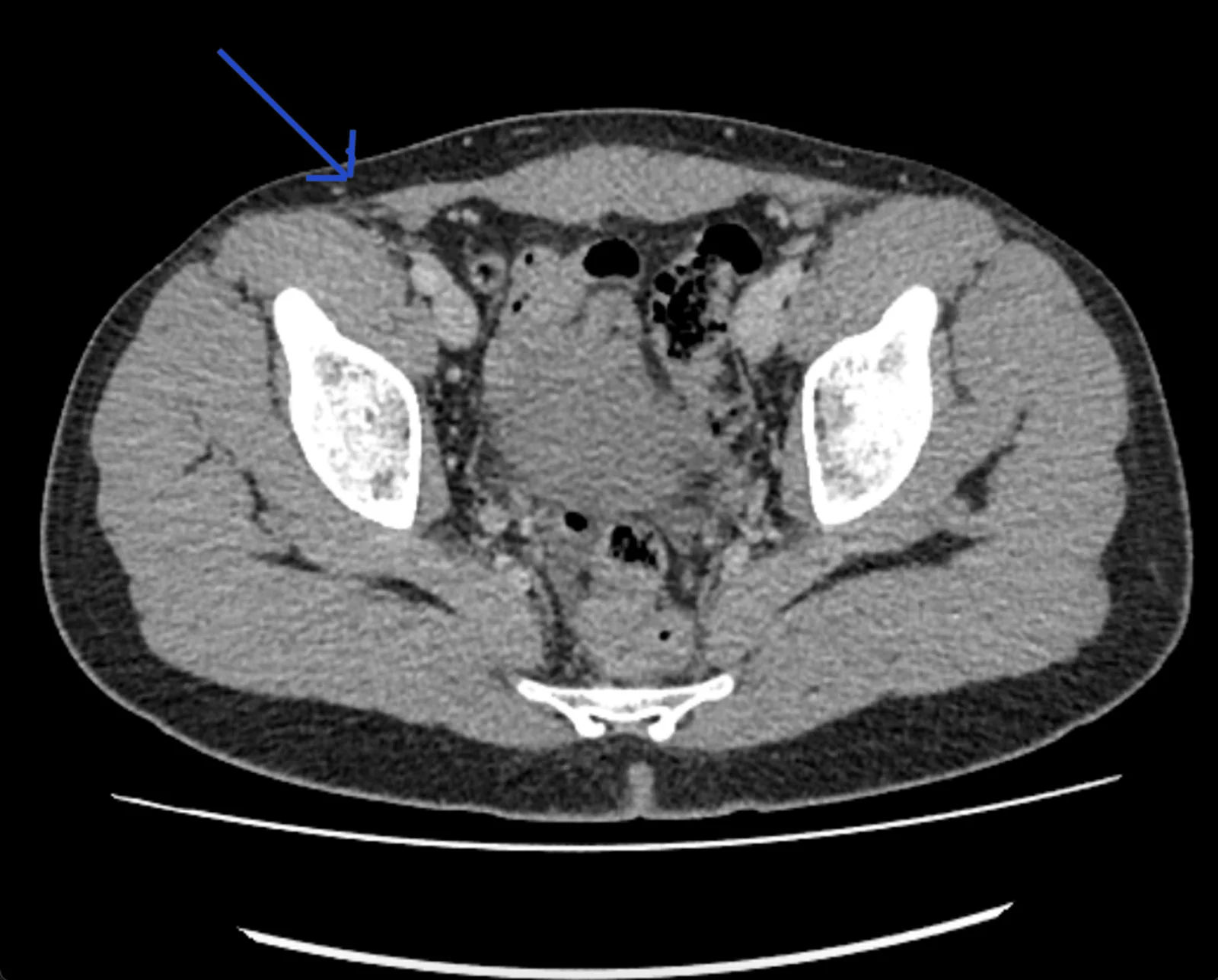
Initial CTAP imaging CTAP: CT of the abdomen and pelvis CTAP with intravenous contrast, Day 0. Axial section through the lower abdomen. No acute pancreatitis or cholecystitis was identified, and the appendix was not visualised on this study. Minimal pelvic free fluid and subcentimetre mesenteric nodes, considered reactive, were present, together with uncomplicated colonic diverticulosis. No cause for the presenting symptoms was found

He returned on Day 2 with worsening symptoms and rigors. The white cell count was 13.8 x 10⁹/L with a neutrophilia of 11.2 x 10⁹/L and a C-reactive protein of 8.6 mg/L. Chest radiography was normal. He was admitted under general medicine and treated with intravenous co-amoxiclav, morphine, ondansetron, and paracetamol. The antibiotic was given for a possible concurrent urinary tract infection, with urine culture still pending at discharge. He was discharged on Day 3 with working diagnoses of possible CHS and coronary artery spasm and attended the same day again, vomiting profusely and in distress. He received intravenous haloperidol 2.5 mg and was admitted again. CVS was recorded as a suspected diagnosis from this point onwards.

Abdominal ultrasound on Day 5 showed no gallstones, a normal common bile duct measuring 4 mm, and no ultrasound evidence of appendicitis in the RIF. Troponin I was 3 ng/L and D-dimer was below 0.27 mg/L fibrinogen equivalent units. The ECG showed sinus rhythm with normal PR and QTc intervals. He attended again on Day 20 with worsening vomiting and abdominal pain despite his medication. The abdomen was soft with mild to moderate left lower quadrant and central tenderness, and he was treated with intravenous antiemetics, fluids, and analgesia.

On Day 72, he attended with chest pain that had started at work and settled on its own before he was assessed. Troponin was below 2.3 ng/L. Suspected oesophageal spasm was documented, and oesophagogastroduodenoscopy (OGD) was suggested, although he had not attended an earlier gastroenterology outpatient appointment.

On Day 98, he presented with severe abdominal pain, shivering, and repeated vomiting. He was still using cannabis at that point. The abdomen was soft and mildly tender. The white cell count was 16.1 x 10⁹/L with a neutrophilia of 13.8 x 10⁹/L. He was admitted and transferred to the gastroenterology ward. The white cell count rose to 16.9 x 10⁹/L with a neutrophilia of 15.4 x 10⁹/L on Day 99

On Day 100, CT of the abdomen and pelvis was repeated and reported in direct comparison with the Day 0 study. The appendix was mildly thickened relative to the previous scan, with a small volume of pelvic free fluid. There was no fat stranding, no free air, and no feature of complication. The conclusion was mild acute appendicitis (Figure 2).

**Figure 2:**
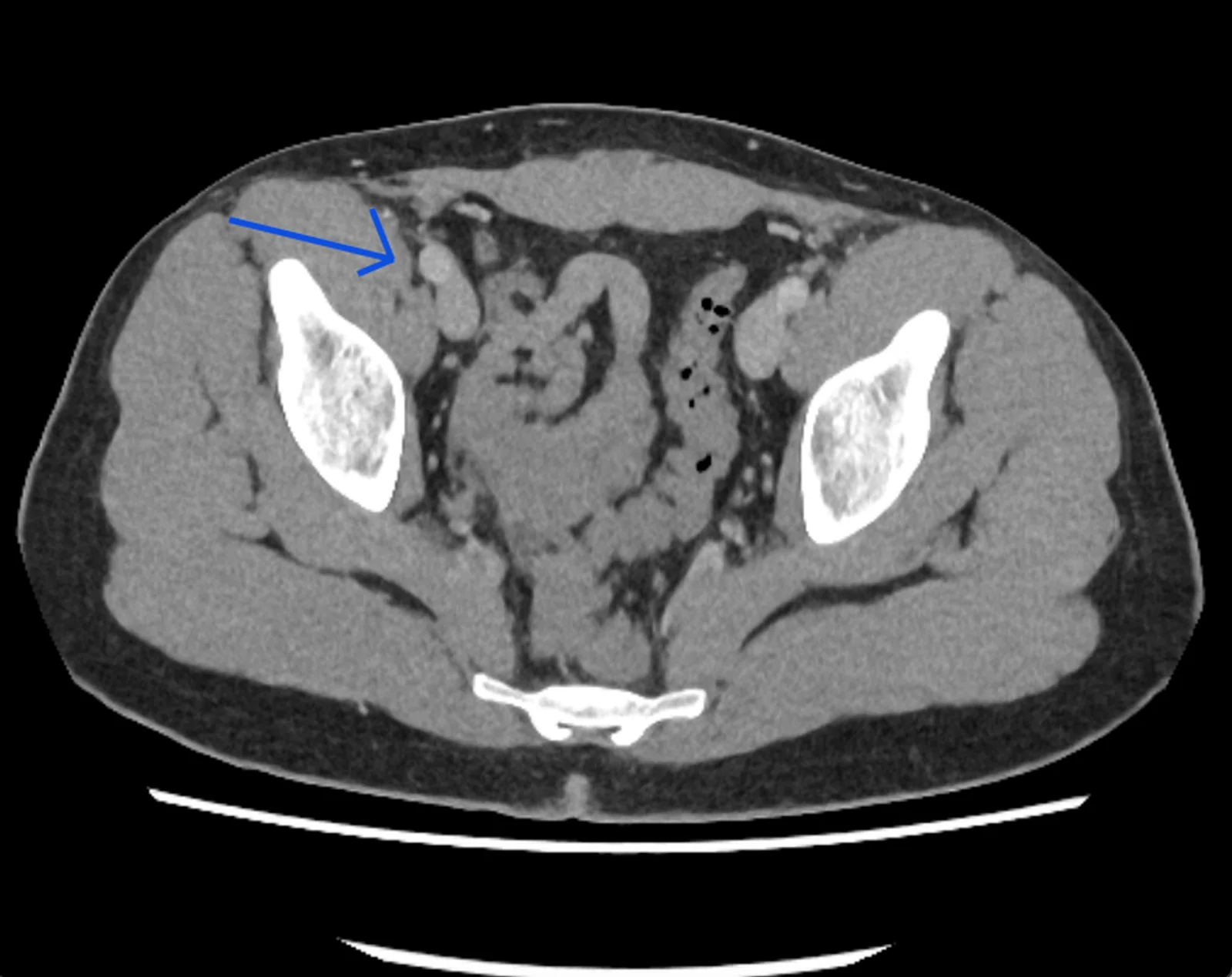
CTAP March 2026 CTAP: computed tomography of the abdomen and pelvis CTAP with intravenous contrast, reported in direct comparison with the Day 0 study. The appendix is mildly thickened relative to the previous scan, with a small volume of pelvic free fluid. There is no periappendiceal fat stranding, no faecolith, and no free air. The study was reported as showing mild acute appendicitis without features of complication

CT at the time of the appendiceal finding

The appendix was reported as mildly thickened on this study, appeared mildly dilated and injected at operation six days later, and was histologically normal. Table 2 summarises imaging across the clinical course.

**Table 2:** Imaging performed across the clinical course, in chronological order CT: computed tomography Consultant radiologists reported all studies. The Day 100 study was explicitly compared with the Day 0 study, and the appendiceal finding was described as a change relative to that scan rather than as an isolated observation

Date	Modality	Findings	Conclusion
Day 0	CT abdomen and pelvis with intravenous contrast	No acute pancreatitis or cholecystitis. Appendix not visualised. Subcentimetre reactive mesenteric nodes. Minimal pelvic free fluid. Uncomplicated colonic diverticulosis. Normal lower thorax	No cause identified for the presenting symptoms
Day 5	Ultrasound abdomen	Normal liver and gallbladder with no gallstones. Common bile duct 4 mm. Normal spleen, kidneys, and aorta. Pancreas partially obscured by bowel gas. No ultrasound evidence of appendicitis in the right iliac fossa	No pathology demonstrated
Day 100	CT abdomen and pelvis with intravenous contrast, reported in comparison with the reported in comparison with the Day 0 study	Appendix slightly thickened compared with the prior study. Small volume of pelvic free fluid. No fat stranding. No free air. All other organs unremarkable	Mild acute appendicitis. No features of complication
Day 138	CT abdomen and pelvis with intravenous contrast	Change consistent with previous appendicectomy. No free air, free fluid, or collections. All organs unremarkable. No lymphadenopathy. Normal lung bases	No acute pathology within the abdomen

The surgical team reviewed him on the same day. The abdomen remained soft without generalised peritonitis and operation was initially deferred. Conservative management was commenced with intravenous co-amoxiclav, metronidazole, opioid analgesia, antiemetics, and intravenous fluids. His symptoms did not settle. Laparoscopic appendicectomy was performed on Day 106 following consultant discussion and with his agreement.

During the operation, the appendix was mildly dilated and injected. There was no perforation, abscess, or collection, and only minimal clear free fluid in the pelvis. A thorough laparoscopy found a normal liver, gallbladder, stomach, small bowel from the terminal ileum to the duodenojejunal flexure, colon, rectum, bladder, and pelvis. Three ports were used: a 10 mm infraumbilical port placed by open technique and 5 mm ports in the left iliac fossa and suprapubic regions. The mesoappendix was divided to the base of the appendix, two endoloops were applied, and the appendix was transected and retrieved in a bag through the umbilical port for histopathology. Haemostasis was secured, and there was no iatrogenic injury and no operative complication. The American Society of Anesthesiologists (ASA) grade was 1, blood loss was under 100 mL, prophylactic co-amoxiclav was given, and enoxaparin was prescribed after surgery for venous thromboembolism prophylaxis. He was discharged on Day 107 having recovered without complication, and reported stopping cannabis after the operation.

Histopathology of the resected specimen was reported on Day 128. Macroscopically, the vermiform appendix and attached mesoappendix measured 70 x 33 x 9 mm; the lumen was expanded by bile-stained material; no faecolith was identified; and there was no macroscopic inflammation. Microscopically, the specimen was a normal appendix vermiformis with no inflammation, dysplasia, or malignancy.

On Day 138, one month after surgery, he returned with a four-hour history of acute diffuse abdominal pain and continuous vomiting, which he described as approximately the sixth such episode since December 2025. He confirmed that he had stopped using cannabis. Examination showed diffuse abdominal tenderness without peritonism, and the port sites had healed with no sign of infection. The white cell count was 17.3 x 10⁹/L with a neutrophilia of 15.1 x 10⁹/L and a C-reactive protein of 1.5 mg/L. CTAP performed the same day showed changes consistent with previous appendicectomy, with no free air, no free fluid, no collection, and no acute pathology within the abdomen (Figure 3).

**Figure 3:**
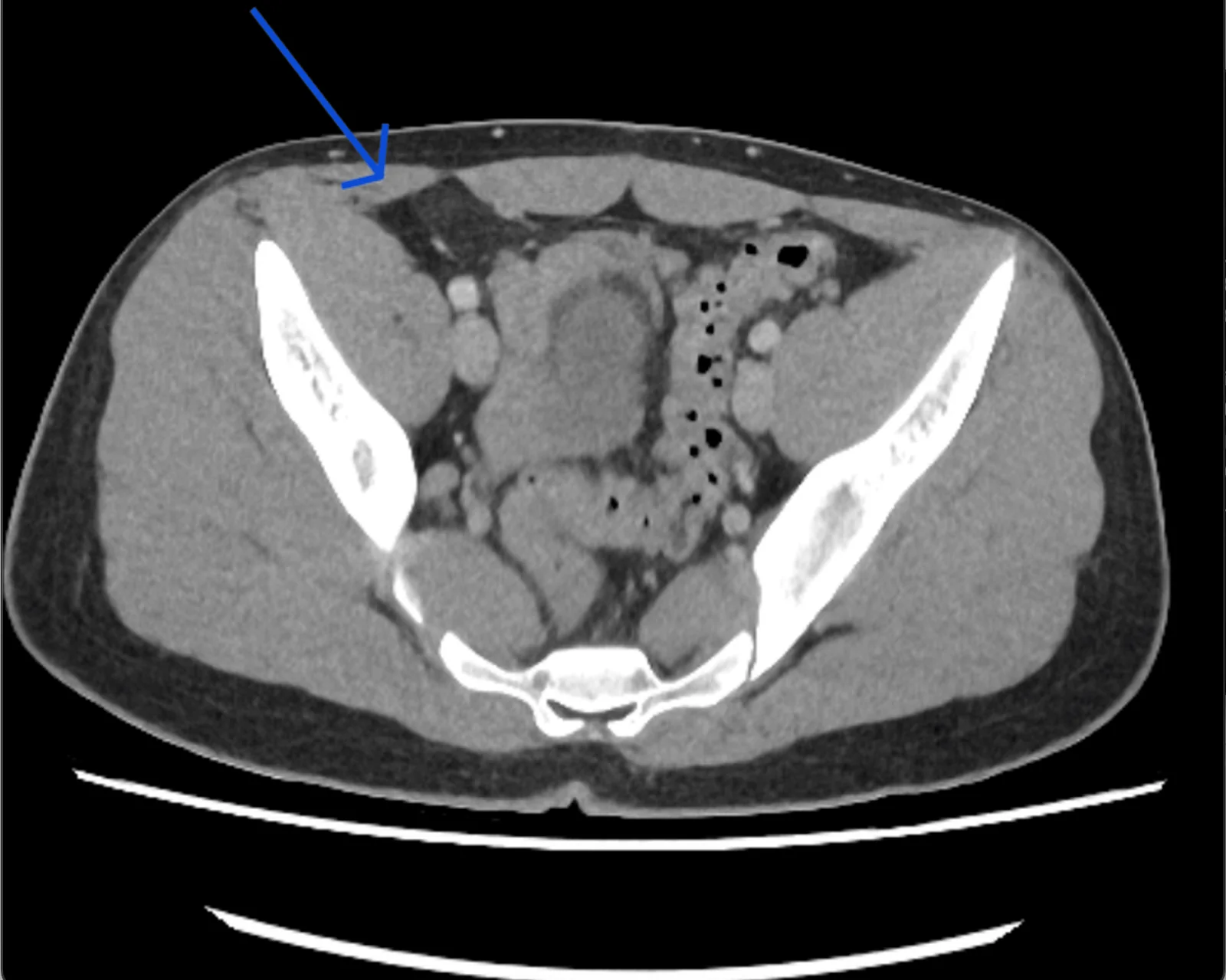
CT after appendicectomy CTAP: computed tomography of the abdomen and pelvis CTAP with intravenous contrast, Day 138, one month after laparoscopic appendicectomy and on the day of a further presentation with pain and vomiting identical in character to those before surgery. There is change consistent with previous appendicectomy, with no free air, no free fluid, and no collection. All other organs are unremarkable, there is no lymphadenopathy, and the lung bases are normal

CTAP with intravenous contrast, Day 138, one month after laparoscopic appendicectomy and on the day of a further presentation with pain and vomiting identical in character to those before surgery. There is change consistent with a previous appendicectomy, with no free air, free fluid, or collection. All other organs are unremarkable; there is no lymphadenopathy, and the lung bases are normal.

He was admitted in May 2026 (Day 142-147). Gastroenterology review identified no acute gastroenterological problem. Faecal calprotectin and blood tests for familial Mediterranean fever were requested, and an urgent outpatient echocardiogram and cardiology referral were arranged given the continuing chest pain and the family history. A cardiology referral note recorded that the earlier Holter monitor had captured a maximum heart rate of 197 beats per minute. He was free of pain and had not vomited for two days before discharge, and no medication was prescribed. The erythrocyte sedimentation rate on Day 147 was 2 mm/hr and serum amyloid A was below 2.0 mg/L. Faecal calprotectin measured on Day 149 was 159 microg/g.

Echocardiography on day 177 showed a normal left ventricular cavity size with an ejection fraction of 60% measured in three dimensions, normal right ventricular size and function, normal atria with an intact interatrial septum, structurally normal valves with only trivial regurgitation, a low probability of pulmonary hypertension, and no pericardial effusion

Renal function was preserved throughout, with an eGFR consistently above 90 mL/min/1.73 m². Serum amylase was normal on every occasion tested, ranging from 59 to 103 U/L, and the coagulation screen was normal whenever performed. There was no biochemical evidence of pancreatitis or biliary obstruction at any point. A pattern of isolated, fluctuating hyperbilirubinaemia was present throughout the course, peaking at 43 µmol/L on day 186, with trivial and inconsistent elevations of alanine aminotransferase, a normal alkaline phosphatase throughout, and well-preserved albumin. This pattern has been present since his first documented blood tests in July 2025. Fractionated bilirubin was requested in June 2026 but had not been reported at the time of writing.

On day 186, he discharged himself from a further emergency attendance with abdominal pain and vomiting. He was seen in the gastroenterology outpatient clinic in June 2026 and placed on the urgent waiting list for OGD. The diagnosis remained unresolved, and investigations were ongoing at the time of writing.

## Discussion

Three assessments of the same appendix did not agree. CT reported mild acute appendicitis. The operating surgeon found a mildly dilated and injected appendix. The pathologist found no inflammation. All three are documented in the clinical record.

CTAP is an accurate investigation for acute appendicitis, with pooled sensitivity approaching 97% and specificity around 96% in adults [3]. Those figures come from studies using histopathology as the reference standard. In daily practice, the radiologist reads the scan alongside clinical information, and a study reported that, in a patient with abdominal tenderness and a raised white cell count, the scan is read against a higher pretest probability than in a blinded research comparison. The main finding on the March 2026 study was a comparative one: a slightly thickened appendix set against a scan performed three months earlier. There was no fat stranding, no faecolith, and only a small volume of free fluid. Findings of this kind are equivocal, and we could not identify an accepted threshold for determining when they justify initiating operation in a patient with recurrent symptoms and a previously negative scan.

A mildly dilated and injected appendix with minimal free fluid is not diagnostic of appendicitis either. In a series of 1,035 appendicectomies, the sensitivity of the surgeon's assessment at operation was 95.13%, but its specificity in adults was only 40.58%, and the authors advised that clinical variables and not macroscopic appearance alone should guide the decision to proceed [12]. Injection and mild dilatation can result from reactive change due to adjacent inflammation, functional bowel disturbance from repeated vomiting, or normal variation. No other gross intra-abdominal or pelvic abnormality was identified during the thorough laparoscopy.

Histology is the most definitive of the three assessments. A consultant histopathologist reporting no inflammation, dysplasia, or malignancy establishes that the appendix was not inflamed. Rates of histologically normal appendix after appendicectomy vary widely with patient selection, imaging availability, and operative threshold, and reached 20.6% in a UK multicentre series [5]. Conservative management with antibiotics was tried first. That is a defensible starting position, supported by randomised evidence that most patients with uncomplicated appendicitis can be managed without operation, although a substantial proportion recur within five years [13,14]. When conservative management failed, operating on the available CT evidence was reasonable. The histology does not make that decision wrong at the time it was taken. However, six days of antibiotic treatment occurred between CT and appendicectomy, so treated or resolving appendicitis cannot be excluded as an explanation for the subsequent normal histology. The recurrence of the same symptoms after surgery indicates that appendicectomy did not resolve the underlying recurrent syndrome. 

CVS and CHS remain the most plausible working diagnoses, and both are diagnoses of exclusion under Rome IV [6,7]. The criteria for CHS require resolution of vomiting after sustained cannabis abstinence [7,8]. He reported stopping cannabis after his March 2026 operation, yet attended in April, May, and June with the same presentation and discharged himself in June. Symptoms persisting after cessation do not exclude CHS, since resolution can take weeks to months, but the diagnosis cannot be confirmed on what we have. CVS remains possible, either on its own or overlapping with CHS [9,10].

The faecal calprotectin of 159 microg/g raises a separate question. In a pooled analysis of 17 studies and 1,956 adults, faecal calprotectin separated inflammatory bowel disease (IBD) from irritable bowel syndrome (IBS) with a pooled sensitivity of 85.8% and a specificity of 91.7%, and sensitivity was better at a threshold of or below 50 microg/g. At a disease prevalence of 1%, the negative predictive value was 99.8% while the positive predictive value was only 9% [15]. A single mildly elevated value therefore carries limited diagnostic weight in isolation. Set against a family history of ulcerative colitis in two maternal relatives, it is still enough to make formal exclusion of IBD worthwhile, and further gastroenterological assessment should address that. Testing for FMF is also reasonable. The condition presents with inflammatory attacks of fever and polyserositis that settle without treatment and are accompanied by a raised acute phase response, and it is increasingly recognised outside the eastern Mediterranean populations in which it is most prevalent [16].

The isolated fluctuating hyperbilirubinaemia present from his first blood tests in July 2025 fits best with Gilbert's syndrome, or with haemoconcentration during acute vomiting. Gilbert's syndrome is the commonest cause of moderate unconjugated hyperbilirubinaemia. It is diagnosed by excluding haemolysis and liver disease, and is characteristically associated with normal aminotransferases and alkaline phosphatase, with bilirubin that rises with fasting, intercurrent illness, and dehydration [17]. All three applied to this patient repeatedly. No structural biliary abnormality was found on any ultrasound or CT. The fractionated bilirubin requested in June 2026 should clarify this.

The chest pain that ran alongside the abdominal presentations is still not fully explained. The June 2026 echocardiogram excludes hypertrophic cardiomyopathy, which had been queried based on the family history, and excludes structural cardiac disease more generally. Serial ECGs were normal after the single early finding attributed to a normal variant. The formal Holter report was not available in the records we reviewed and should be seen by cardiology before the cardiac question is closed. Oesophageal spasm was raised as a possibility in February 2026 and would also be addressed by the planned OGD.

What concerns us most is how a functional label behaves once it is in the record. CVS was recorded as a suspected diagnosis in December 2025, the day he came back to the emergency department having been discharged only hours earlier, and CHS was recorded as a discharge diagnosis around the same time. Both were applied before gastroenterology was involved, before endoscopy was considered, and before the exclusions required by Rome IV were completed. Every team that saw him afterwards had to make a deliberate decision to look past those labels. Some did. The March 2026 CT was requested because the clinical picture had changed. Even so, an early functional diagnosis narrowed the scope of what was investigated afterwards [11]. When a patient returns repeatedly with the same symptoms despite treatment, the diagnosis itself needs review, not another round of the same management.

## Conclusions

CT findings interpreted as mild appendicitis, the macroscopic appearance at operation, and histopathology can point in three different directions in the same patient. A histologically normal appendix after appendicectomy does not answer the diagnostic question. It leaves it open. The recurrent vomiting and abdominal pain in this patient began before the CT finding, continued after surgery, and were still unexplained at the time of writing. CT evidence of mild appendicitis in a patient who carries a functional label and has a history of recurrent vomiting should be interpreted alongside the whole clinical picture and not treated as decisive. Histology from every resected appendix should be reviewed and passed back to the team responsible for follow-up. Where symptoms persist after appendicectomy and the specimen is histologically normal, the differential diagnosis should be reconsidered rather than continuing symptomatic treatment alone.
